# Increased In Vitro Intercellular Barrier Function of Lung Epithelial Cells Using Adipose-Derived Mesenchymal Stem/Stromal Cells

**DOI:** 10.3390/pharmaceutics13081264

**Published:** 2021-08-16

**Authors:** Mitsutoshi Ishii, Tomoshi Tsuchiya, Ryoichiro Doi, Yoichi Morofuji, Takashi Fujimoto, Hideki Muto, Takashi Suematsu, Ryoichi Mori, Keitaro Matsumoto, Takuro Miyazaki, Koichi Tomoshige, Hironosuke Watanabe, Mayumi Iwatake, Takeshi Nagayasu

**Affiliations:** 1 Department of Surgical Oncology, Nagasaki University Graduate School of Biomedical Sciences, 1-12-4 Sakamoto, Nagasaki 852-8523, Japan; mi14132104@yahoo.co.jp (M.I.); ryoichiro_doi_japan@msn.com (R.D.); kmatsumo@nagasaki-u.ac.jp (K.M.); miyataku@nagasaki-u.ac.jp (T.M.); tomoshige@nagasaki-u.ac.jp (K.T.); hironosuke3689@yahoo.co.jp (H.W.); iwatake@nagasaki-u.ac.jp (M.I.); 2 Department of Neurosurgery, Nagasaki University Hospital, 1-7-1 Sakamoto, Nagasaki 852-8501, Japan; morofujiyoichi@gmail.com (Y.M.); t.fujimototakashi@gmail.com (T.F.); 3 Biomedical Research Support Center, Nagasaki University School of Medicine, 1-12-4 Sakamoto, Nagasaki 852-8523, Japan; hidemuto@nagasaki-u.ac.jp; 4 Division of Electron Microscopy, Nagasaki University Graduate School of Biomedical Sciences, 1-12-4 Sakamoto, Nagasaki 852-8523, Japan; suematsu@nagasaki-u.ac.jp; 5 Department of Pathology, School of Medicine and Graduate School of Biomedical Sciences, Nagasaki University, 1-12-4 Sakamoto, Nagasaki 852-8523, Japan; ryoichi@nagasaki-u.ac.jp

**Keywords:** mesenchymal stem/stromal cell, alveolar epithelial cell, barrier function, air-blood barrier, transepithelial electrical resistance, acute respiratory distress syndrome

## Abstract

With the emergence of coronavirus disease-2019, researchers have gained interest in the therapeutic efficacy of mesenchymal stem/stromal cells (MSCs) in acute respiratory distress syndrome; however, the mechanisms of the therapeutic effects of MSCs are unclear. We have previously reported that adipose-derived MSCs (AD-MSCs) strengthen the barrier function of the pulmonary vessels in scaffold-based bioengineered rat lungs. In this study, we evaluated whether AD-MSCs could enhance the intercellular barrier function of lung epithelial cells in vitro using a transwell coculture system. Transepithelial electrical resistance (TEER) measurements revealed that the peak TEER value was significantly higher in the AD-MSC coculture group than in the AD-MSC non-coculture group. Similarly, the permeability coefficient was significantly decreased in the AD-MSC coculture group compared to that in the AD-MSC non-coculture group. Immunostaining of insert membranes showed that zonula occuldens-1 expression was significantly high at cell junctions in the AD-MSC coculture group. Moreover, cell junction-related gene profiling showed that the expression of some claudin genes, including claudin-4, was upregulated in the AD-MSC coculture group. Taken together, these results showed that AD-MSCs enhanced the barrier function between lung epithelial cells, suggesting that both direct adhesion and indirect paracrine effects strengthened the barrier function of lung alveolar epithelium in vitro.

## 1. Introduction

End-stage bacterial/viral infections induce an excessive and unusual host immune response, often accompanied by excessive production of inflammatory cytokines, such as interleukin-2 (IL-2), IL-6, IL-7, granulocyte colony-stimulating factor, interferon gamma-induced protein 10, monocyte chemotactic protein 1, macrophage inflammatory protein 1 alpha, and tumor necrosis factor α (TNF-α), resulting in pulmonary edema and subsequent acute lung injury/acute respiratory distress syndrome (ALI/ARDS) [1]. Once a patient develops ALI/ARDS, treatment is limited, and the mortality rate for patients with ARDS is approximately 35–46% [2]. In ARDS, disruption of the alveolar epithelial-endothelial interface results in the discharge of inflammatory exudates into the alveoli, reduced lung compliance, and impaired gas exchange [3]. Therefore, there is a need to introduce appropriate therapies that can suppress the cytokine storm and induce the repair function of the lungs.

ALI/ARDS is typically treated with anti-inflammatory adrenal steroids and antibacterial agents. However, recent studies have highlighted the potential applications of mesenchymal stem/stromal cells (MSCs). MSCs are cells that have the ability to differentiate into mesenchymal cells, such as osteoblasts, adipocytes, myocytes, and chondrocytes. These cells have attracted attention as a source of cells for cellular and regenerative medicine because of their low risk of tumor formation, immune regulation, wound healing, and differentiation potential, including nerve regeneration. MSCs are known to secrete soluble factors, such as IL-6, IL-10, transforming growth factor β, indoleamine 2,3-dioxygenase, intercellular adhesion molecule, TNF-stimulated gene protein-6, and prostaglandin E2, which regulate immune cells [4,5,6,7,8,9]. In addition, comparisons based on the cellular origin of MSCs are actively performed, because their surface phenotypes and chemokine/cytokine gene expression vary with the cell origin [10,11]. For example, compared to bone marrow-derived MSCs (BM-MSCs), adipose-derived MSCs (AD-MSCs) highly induce the release of IL-6, IL-10, and TGF-β in the culture medium [10]. AD-MSCs exhibit the shortest time for proliferation, adipogenesis, and osteogenic differentiation among the several types of MSCs, including those of placental and umbilical cord origins [11].

Several reports have indicated that MSCs can strengthen the barrier function of the vascular system [12,13,14]. Hepatocyte growth factor (HGF), angiopoietin-1 (Ang-1), and keratinocyte growth factor (KGF) secreted from MSCs have been reported to improve vascular endothelial barrier function [15,16,17,18,19]. In an ex vivo study, we reported that AD-MSCs differentiate into pericytes and induce pulmonary vessel maturation in rat bioengineered lungs using a decellularized lung scaffold [20]. Orthotopic transplantation of the bioengineered lungs revealed that pulmonary edema was clearly suppressed, suggesting that the endothelial intercellular barrier function was enhanced by the addition of AD-MSCs.

However, the barrier system of the lung alveoli consists of an alveolar epithelial-endothelial interface, which indicates that the epithelial barrier function is also required to maintain lung alveolar gas-exchange function. In fact, the epithelial barrier is much less permeable than the endothelial barrier [21]. Even when endothelial permeability is normal, damage to the alveolar epithelium is sufficient for the formation of pulmonary edema [22]. Although MSCs have been used clinically for the treatment of ALI/ARDS, the effects of these cells on the barrier function of lung epithelial cells have not been investigated.

Therefore, in the current study, we analyzed the effects of AD-MSCs on lung epithelial intercellular barrier function in vitro. The transwell coculture system simulated the alveolar epithelium and demonstrated the beneficial effects of MSCs on lung alveolar damage.

## 2. Materials and Methods

### 2.1. Isolation and Culture of AD-MSCs

Inguinal adipose tissue was obtained from young adult male Fischer 344 rats (8 weeks old; CLEA, Tokyo, Japan). AD-MSCs were isolated from adipose tissue according to the method described by Zuk et al. [23], with minor modifications. Briefly, the washed adipose tissue was cut into small pieces and digested with collagenase (Celase, Cytori Therapeutics, Tokyo, Japan) in phosphate-buffered saline (PBS; Wako, Osaka, Japan) for 30 min in a shaking water bath at 37 °C. Collagenase was subsequently inactivated with an equal volume of PBS/5% bovine serum albumin (BSA). The mature adipocyte fraction was separated from the stromal vascular fraction by centrifugation (400× *g*, 10 min), and the resulting cell pellets were resuspended in Dulbecco’s modified Eagle medium: Nutrient Mixture F-12 (DMEM/F-12; Gibco, Gaithersburg, MD, USA). After successive filtration through 100- and 40-µm cell strainers, the freshly isolated cells were cultured in DMEM/F-12 with 10% fetal bovine serum (FBS; Gibco) and 1% penicillin (100 IU/mL)/streptomycin (100 μg/mL; Gibco)/amphotericin B (0.25 μg/mL; Sigma-Aldrich, St. Louis, MO, USA) at 37 °C in an atmosphere containing 5% carbon dioxide. The culture medium was changed every 3 days, and the cells were passaged after reaching 80–90% confluence. Cells were used for experiments at passages 2–3.

### 2.2. Isolation and Culture of BM-MSCs

We used three-week-old male Fischer 344 rats to isolate BM-MSCs. Briefly, the femurs were detached from the hindlimbs, and the muscles were removed. BM cells were isolated by flushing the femoral cavity with PBS and culturing the obtained cells in DMEM/F-12 with 10% FBS and 1% penicillin (100 IU/mL)/streptomycin (100 μg/mL)/amphotericin B (0.25 μg/mL). The culture medium was changed every 3 days, and the cells were passaged after reaching 80–90% confluence. Cells were used for experiments at passages 2–3.

### 2.3. Isolation of Lung Cells

Fisher 344 rats (3–4 weeks old) were exposed to general anesthesia. The pulmonary artery was cannulated and perfused with 25 mL PBS containing 50 U/mL heparin (Mochida, Tokyo, Japan) and 1 μg/mL sodium nitroprusside (Sigma-Aldrich), and the lungs were removed. The alveoli were washed twice by injecting and draining PBS from the trachea. The alveoli were then filled with solution A (DMEM/F-12 + 2.5% HEPES (Wako) + 4.5 U/mL elastase (Worthington, Lakewood, NJ, USA) + 0.02 mg/mL DNase I (Sigma-Aldrich)), placed in a flask containing solution A, and shaken in a shaker at 37 °C for 45 min. The peripheral two-thirds of the lungs were then minced, placed in a new flask with solution A, and shaken for 15 min in a shaker at 37 °C. After shaking, a solution containing FBS (DMEM/F-12 + 2.5% HEPES + 50% FBS) was added, and the reaction was stopped by cooling on ice for 5 min. After successive filtration through 100- and 70-µm cell strainers, the lung cells were separated by centrifugation (300× *g*, 5 min), and the resulting cell pellets were resuspended in DMEM/F-12.

### 2.4. Flow Cytometry Analysis and Sorting of Alveolar Epithelial Type II (ATII) Cells

For flow cytometry analysis of lung cells, the pellet obtained from lung tissue was resuspended in fluorescence-activated cell sorting (FACS) buffer (45 mL PBS (pH 7.2) containing 5 mL of 5% FBS and 1 mL of 100 mM ethylenediaminetetraacetic acid (EDTA; Nacalai Tesque, Kyoto, Japan)). Anti-RT1-40 antibodies (1:100; cat. no. TB-11ART1-40; Terrace Biotech, San Francisco, CA, USA) and anti-RT2-70 antibodies (1:100; cat. no. TB-44ART2-70; Terrace Biotech) were added as primary antibodies. Mouse IgG1 kappa isotype control (1:100; cat. no. 14-4714-82; Thermo Fisher, Waltham, MA, USA) and mouse IgG3 isotype control (1:100; cat. no. 14-4742-82; Thermo Fisher) were used as isotype controls. The mixture was incubated on ice for 30 min. After centrifugation (300× *g*, 5 min, 4 °C) and washing twice, allophycocyanin (APC)-conjugated anti-mouse IgG1 antibodies (1.25:100; cat. no. 406610; BioLegend, San Diego, CA, USA) and fluorescein isothiocyanate-conjugated anti-mouse IgG3 (2:100; cat. no. 1191-02; Southern Biotech, Birmingham, AL, USA) were added as secondary antibodies and incubated for 30 min. After centrifugation (300× *g*, 5 min, 4 °C) and washing twice, the cells were resuspended in FACS buffer, and flow cytometry analysis was conducted using a BD FACS Aria II.

For isolation of ATII cells, lung cell pellets were resuspended in FACS buffer, and anti-RT2-70 antibodies (3:100) were added as the primary antibody (mouse IgG3 isotype control (1:100) was used as an isotype control). The samples were then incubated on ice for 30 min. After centrifugation (300× *g*, 5 min, 4 °C) and washing twice, Alexa Fluor647 goat anti-mouse IgG (1:200; cat. no. A-21235; Thermo Fisher) was added as a secondary antibody and incubated for 30 min. After centrifugation (300× *g*, 5 min, 4 °C) and washing twice, the cells were resuspended in FACS buffer, and ATII cells were isolated from the lung cells using a BD FACS Aria II.

### 2.5. Transepithelial Electrical Resistance (TEER) Measurement

#### 2.5.1. Comparison of AD-MSCs and BM-MSCs

For this assay, 1 × 10^5^ lung cells were seeded on the apical side of insert membranes (Millicell Hanging Cell Culture Insert, PET 0.4 µm, 24-well; Merck Millipore, Burlington, MA, USA) with a pore size of 0.4 µm and a growth area of 0.33 cm^2^. AD-MSCs or BM-MSCs (1 × 10^5^) were seeded on the bottom wells of a 24-well plate to coculture the two cell types in a non-contact manner. Lung cells cultured alone were designated as the non-coculture group. TEER of the three groups was measured every 24 h using Millicell ERS-2 (Merck Millipore; *n* = 5). The culture medium was DMEM/F-12 with 10% FBS and 1% penicillin (100 IU/mL)/streptomycin (100 μg/mL)/amphotericin B (0.25 μg/mL), and the medium was changed every other day.

#### 2.5.2. TEER Measurement in AD-MSC Non-Contact Culture

Lung cells (1 × 10^5^) were seeded on the apical side of insert membranes (24 wells, pore size 0.4). AD-MSCs (1 × 10^5^) were seeded on the bottom wells of a 24-well plate to coculture the two cell lines in a non-contact manner. Lung cells cultured alone were designated as the non-coculture group. The TEER of the two groups was measured every 24 h using Millicell ERS-2 (*n* = 3). The culture medium was DMEM/F-12 with 10% FBS and 1% penicillin (100 IU/mL)/streptomycin (100 μg/mL)/amphotericin B (0.25 μg/mL), and the medium was changed every other day.

TEER of the ATII cell monolayer was also measured in the AD-MSC non-contact group and the AD-MSC non-coculture group (*n* = 3).

#### 2.5.3. TEER Measurement in AD-MSC Contact Culture

Lung cells (1 × 10^5^) were seeded on the apical side of the insert membrane (24 well, pore size 0.4). The day before, 3.3 × 10^4^ AD-MSCs were seeded on the basolateral side to coculture the two cell lines in a contact manner. Lung cells cultured alone were designated as the AD-MSC non-coculture group. The TEER of the two groups was measured every 24 h using Millicell ERS-2 (*n* = 3). The medium used was DMEM/F-12 with 10% FBS and 1% penicillin (100 IU/mL)/streptomycin (100 μg/mL)/amphotericin B (0.25 μg/mL). During the culture period, the medium was changed every other day.

The lung cells were replaced with ATII cells, and TEER values were also measured in the AD-MSC contact group and the AD-MSC non-coculture group (*n* = 3).

#### 2.5.4. TEER Measurement Using an Air–Blood Barrier (ABB) Model

ATII cells (1 × 10^5^) were seeded on the apical side of the insert membrane (24 well, pore size 0.4). The day before, 3.3 × 10^4^ rat lung microvascular endothelial cells (RLMVECs; VEC Technologies Inc., Rensselaer, NY, USA) were seeded on the basolateral side for coculture of the two cell lines in a contact manner (the ABB model). AD-MSCs (1 × 10^5^) were seeded on the bottom wells of a 24-well plate for the AD-MSC coculture group. The ABB model cultured without AD-MSCs was designated as the AD-MSC non-coculture group. The TEER of the two groups was measured every 24 h using Millicell ERS-2 (*n* = 3). The medium used was DMEM/F-12 with 10% FBS and 1% penicillin (100 IU/mL)/streptomycin (100 μg/mL)/amphotericin B (0.25 μg/mL), and medium changes were performed every other day.

**Figure 1:**
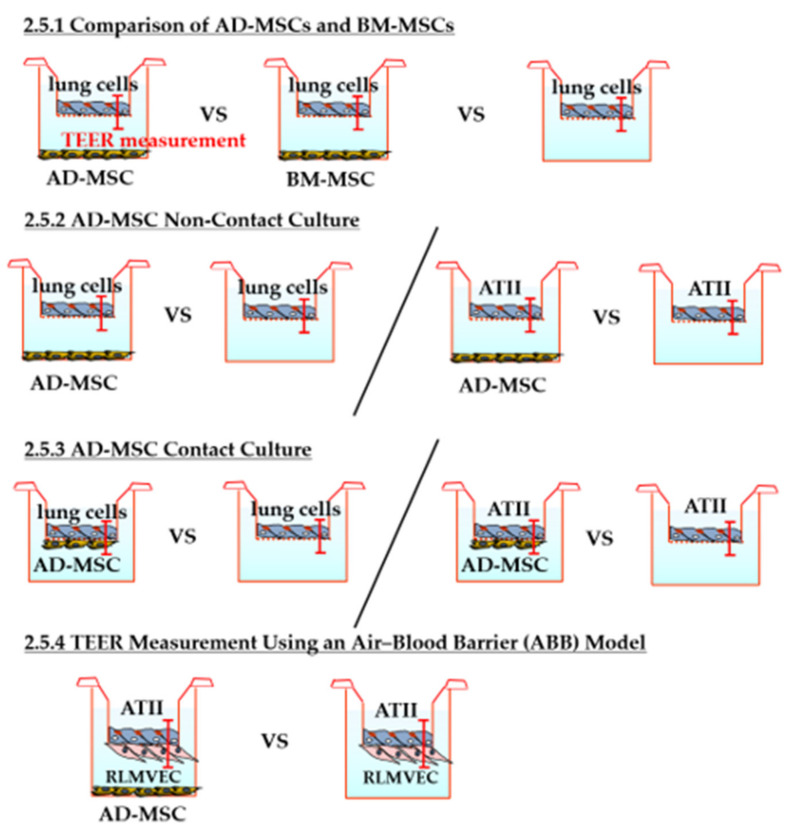
Schematic of the experiment design. TEER, transepithelial electrical resistance; AD-MSC, adipose-derived mesenchymal stem/stromal cell; BM-MSC, bone marrow-derived mesenchymal stem/stromal cell; ATII, alveolar epithelial type II; RLMVEC, rat lung microvascular endothelial cell.

The experimental setup of TEER measurement is shown in Figure 1.

### 2.6. Permeability Assay

To evaluate the permeability of the monolayers, 1 × 10^5^ lung cells were seeded on the apical side of insert membranes (24 wells, pore size 0.4). AD-MSCs (1 × 10^5^) were seeded on the bottom wells of a 24-well plate in the AD-MSC coculture group. Lung cells cultured alone were designated as the AD-MSC non-coculture group. Permeability assays were performed on day 4, according to the methods described by Elbert et al. [24], with minor modifications. Briefly, the lung cell monolayer was washed with prewarmed assay buffer (10 mL D-PBS(+) containing 1 mL of 1 M HEPES, 0.45 g d-glucose (Wako), and 89 mL distilled water). Next, 300 µL assay buffer with 10 µg/mL sodium fluorescein (FluNa, molecular weight 376.28 Da; Wako) ± 16 mM EDTA was added to the apical compartment (donor), and 1000 µL assay buffer was added to the basolateral compartment (acceptor). Immediately after adding the solutions, samples were taken from the donor (100 µL) and the acceptor (100 µL) and transferred into a 96-well plate to measure the starting concentrations. Subsequently, the plates were placed on an orbital shaker (80 rpm) in an incubator at 37 °C, and 100 µL of each sample was collected every 30 min from the basolateral compartment, for 2 h; 100 µL assay buffer was added to refill each well. At the end of the experiment, samples in the 96-well plate were measured with a plate reader at 490 nm excitation and 570 nm emission wavelengths.

The permeability of the monolayers of ATII cells was also measured on day 7 in both the AD-MSC coculture and non-coculture groups.

The apparent permeability coefficient (*Papp*) was calculated from the following equation:

$$Papp=\frac{V}{A\times \left[C\right]apical}\times \frac{\Delta \left[C\right]basolateral}{\Delta t}$$

where *V* is the volume of the basolateral compartment (cm^3^); [*C*] is the concentration of FluNa (µg/mL); *A* is the surface area of the insert membrane (cm^2^); and *t* is the time (s).

### 2.7. Hematoxylin and Eosin (HE) Staining of Insert Membranes

ATII cells previously grown on insert membranes were fixed on day 7 with 4% paraformaldehyde for 30 min at room temperature. Subsequently, the samples were dehydrated using a series of ethanol concentrations (35%, 50%, 70%, 95%, 95%, 100% for 10 min each), followed by treatment with xylene for 10 min. Subsequently, the samples were embedded in paraffin and cut into 5-μm-thick slices the next day. The sections were deparaffinized, rehydrated, stained with Mayer’s hematoxylin (Muto Pure Chemical, Tokyo, Japan) for 5 min and eosin Y (Sigma-Aldrich) for 2 min, and then mounted with malinol (Muto Pure Chemical) after dehydration and permeabilization. Samples were analyzed using light microscopy.

### 2.8. Immunostaining of Insert Membranes

To assess the phenotypic characteristics of ATII cells, ATII cell monolayers cultured on insert membranes were stained with RT1-40, the antibody that binds to rat ATI cells, and RT2-70, the antibody that binds to rat ATII cells. On day 4, the insert membranes were washed with PBS and fixed with 4% paraformaldehyde for 10 min at room temperature. Subsequently, the samples were permeabilized with 0.1% Triton-X (Sigma-Aldrich) for 10 min at room temperature and blocked with 3% BSA overnight at 4 °C. The insert membranes were incubated with specific primary antibodies against RT1-40 (1:150) and RT2-70 (1:150) for 45 min at 37 °C, washed with 0.1% BSA solution, and incubated with APC-conjugated anti-mouse IgG1 antibodies (1:100) or donkey anti-mouse IgG 488 secondary antibodies (1:200; cat. no. ab150105; Abcam, Cambridge, UK) for 45 min at 37 °C. After washing with 0.1% BSA solution, the insert membranes were punched out, placed on a glass slide, and sealed with 4′,6-diamidino-2-phenylindole (DAPI). Observations were performed using a Keyence all-in-one fluorescence microscope.

To stain cell junctional proteins, lung cell monolayers and ATII cell monolayers cultured on insert membranes in the absence or presence of non-contact AD-MSCs were stained for zonula occludens-1 (ZO-1; also known as tight junction protein-1). The lung cell monolayer and the ATII cell monolayer, on days 4 and 7, respectively, were washed with PBS and fixed with 4% paraformaldehyde for 10 min at room temperature. Subsequently, the samples were permeabilized with 0.1% Triton-X for 10 min at room temperature and blocked with 3% BSA overnight at 4 °C. The insert membranes were incubated with specific primary antibodies against ZO-1 (1:50; cat. no. 33-9100; Thermo Fisher) for 45 min at 37 °C. The insert membranes were washed with 0.1% BSA solution and incubated with donkey anti-mouse IgG 488 (1:200) secondary antibodies for 45 min at 37 °C. After washing with 0.1% BSA solution, the insert membranes were punched out, placed on glass slides, and sealed with DAPI. Observations were performed using a Keyence all-in-one fluorescence microscope.

### 2.9. Transmission Electron Microscopy

ATII cells (3 × 10^5^) were seeded on the apical side of insert membranes (24 well, pore size 0.4). AD-MSCs (1 × 10^5^) were seeded on the bottom wells of a 24-well plate in the AD-MSC coculture group. ATII cells cultured alone were designated as the AD-MSC non-coculture group. The insert membranes were fixed in 2.5% glutaraldehyde solution buffered to pH 7.4, with 0.1 M phosphate buffer for 4 h at 4 °C for electron microscope examination on day 7. Postfixation was performed with a 1% osmium tetroxide solution buffered to pH 7.4, with the same buffer for 2 h at 4 °C. Samples were dehydrated in a graded series of ethanol concentrations and embedded in Epon 812.

Ultrathin sections were cut with an ultramicrotome (Ultracut S, Leica, Vienna, Austria) with a diamond knife, double-stained with uranyl acetate and lead nitrate, and observed under an electron microscope (JEM-1200EX; JEOL, Tokyo, Japan) at an accelerating voltage of 80 kV.

### 2.10. RNA Extraction, Reverse Transcription, and Quantitative Polymerase Chain Reaction (qPCR)

ATII cells (1 × 10^5^) were seeded on the apical side of insert membranes (24 well, pore size 0.4). AD-MSCs (1 × 10^5^) were seeded on the bottom wells of a 24-well plate in the AD-MSC coculture group. Lung cells cultured alone were designated as the AD-MSC non-coculture group.

Total RNA was isolated from insert membranes on day 7 using an RNeasy Mini Kit (Qiagen, Hilden, Germany) according to the manufacturer’s instructions. Reverse transcription and complementary DNA (cDNA) synthesis were performed using an RT^2^ First Strand Kit (Qiagen) and 50 ng RNA.

To analyze gene expression related to cell junctions, the RT^2^ Profiler PCR Array Rat Cell Junction PathwayFinder (Qiagen), a commercially available PCR array, was used. This PCR array contained 84 key genes involved in rat cell junctions. Briefly, 102 μL cDNA was mixed with 2× RT^2^ SYBR Green Master Mix (1.35 mL; Qiagen) and RNase-free water to a final volume of 2.7 mL. Each well in the RT^2^ Profiler PCR array plate contained a 25 μL sample. PCR was performed using a Roche LightCycler 480, following the manufacturer’s instructions. Five housekeeping genes were included in the array, which enabled the normalization of data. Fold-changes in expression were calculated using the ΔΔCt method.

### 2.11. Statistical Analysis

The results are presented as means ± standard deviations. Comparisons between two groups were evaluated using unpaired Student’s t-tests. Differences between three or more groups were assessed using one-way analysis of variance, followed by Bonferroni tests. Results with *p*-values less than 0.05 were considered significant. All statistical analyses were performed using JMP Pro 15.0.0 software (SAS Institute Inc., Cary, NC, USA).

## 3. Results

### 3.1. Flow Cytometry Analysis and Sorting of ATII Cells

**Figure 2:**
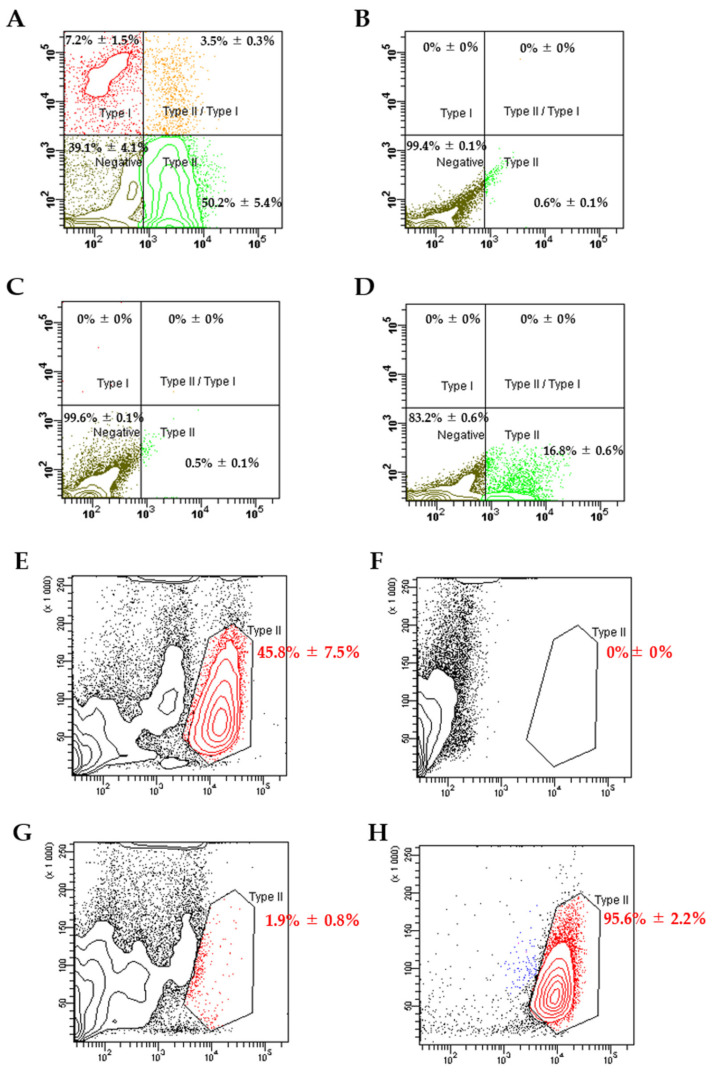
Flow cytometry analysis of extracted lung cells (**A**–**D**) and ATII cell isolation (**E**–**H**). (**A**) Representative flow cytometry analysis of RT1-40 and RT2-70 expression. (**B**) Unstained control. (**C**) Isotype control against RT1-40. (**D**) Isotype control against RT2-70. Nonspecific binding was observed in the isotype control against RT2-70. (**E**) Representative flow cytometry analysis of ATII cell sorting. (**F**) Unstained control. (**G**) Isotype control. (**H**) Data after ATII cell sorting. All data represent means ± standard deviations.

Flow cytometry analysis revealed that 50.2% ± 5.4% of the extracted lung cells were ATII cells, labeled with anti-RT2-70 antibodies, and 7.2% ± 1.5% of the lung cells were alveolar epithelial type I (ATI) cells labeled with anti-RT1-40 antibodies (Figure 2A–D). Some were double-positive cells that had characteristics of ATI and ATII cells. Because a nonspecific reaction was observed in the isotype control against RT2-70 (Figure 2D), the secondary antibody was changed to Alexa Fluor647 goat anti-mouse IgG for isolation of ATII cells (Figure 2E–H).

Additionally, 45.8% ± 7.5% of the obtained lung cells were collected as ATII cells labeled with anti-RT2-70 antibodies by flow cytometry isolation (Figure 2E–H). The cell purity of isolated ATII cells was 95.6% ± 2.2% (Figure 2H).

### 3.2. Morphology and Phenotypic Characteristics of ATII Cells

**Figure 3:**
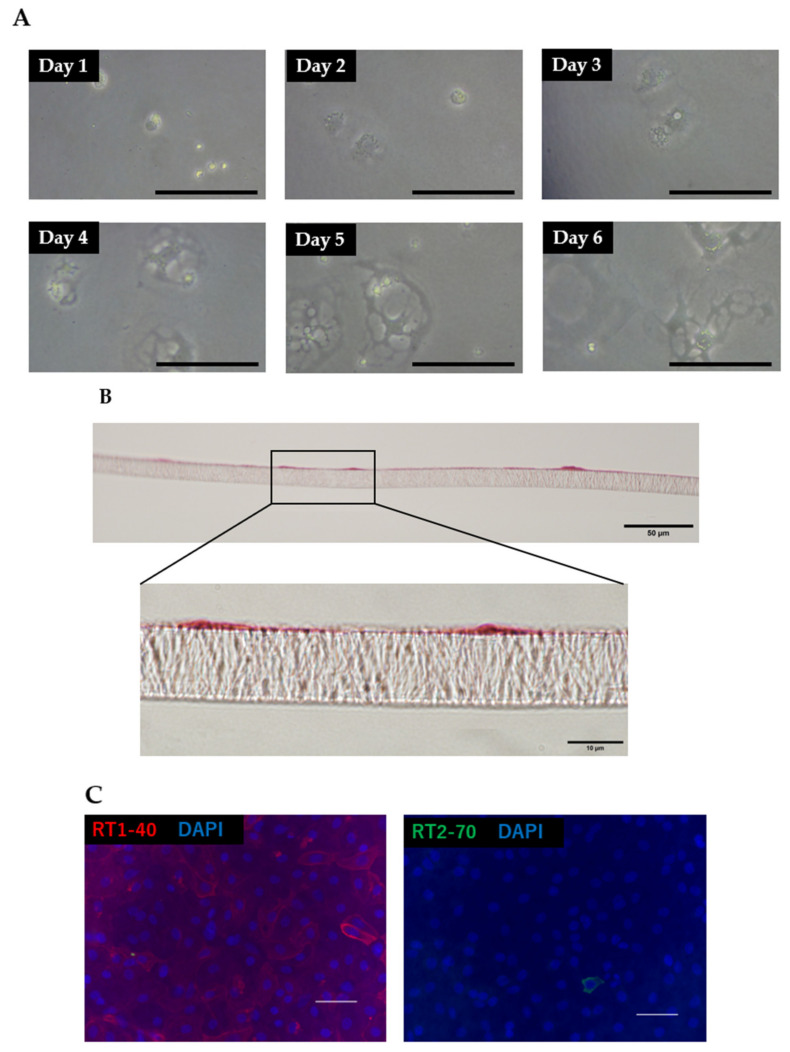
Morphology and phenotypic characteristics of ATII cells. (**A**) ATII cells seeded on tissue culture plastic (scale bar: 100 μm). Notably, attached cuboidal ATII cells were differentiated into flattened ATI-like cells over several days. (**B**) HE staining of insert membranes on day 4 after seeding ATII cells (cross-section). (**C**) Immunofluorescence images of ATII cells stained with RT1-40 (red) and RT2-70 (green) with DAPI (blue) on day 4 after being seeded on insert membranes (scale bar: 50 μm).

When isolated ATII cells were seeded on tissue culture plastic or insert membranes, they gradually changed their shape from smaller, cuboidal cells to larger, flattened cells over several days (Figure 3A). A HE-stained cross-section of the insert membrane on which ATII cells had been seeded 4 days before showed the formation of a monolayer consisting of very thin cells (Figure 3B). Immunostaining on day 4 after the seeding of ATII cells on the insert membranes showed that RT1-40-positive/RT2-70-negative cells accounted for the majority of the cells, suggesting that isolated ATII cells differentiated into ATI cells (Figure 3C).

### 3.3. TEER Measurement

#### 3.3.1. Comparison of AD-MSCs and BM-MSCs

**Figure 4:**
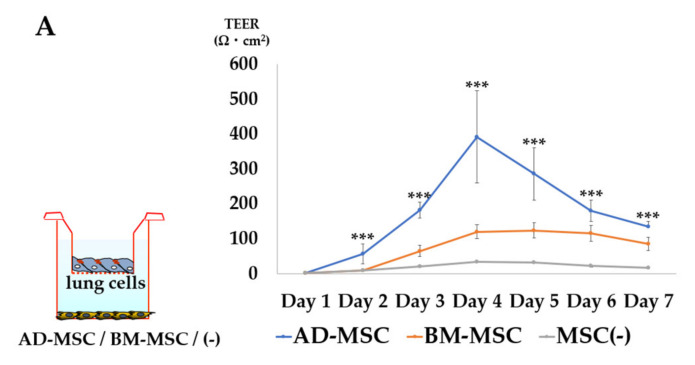

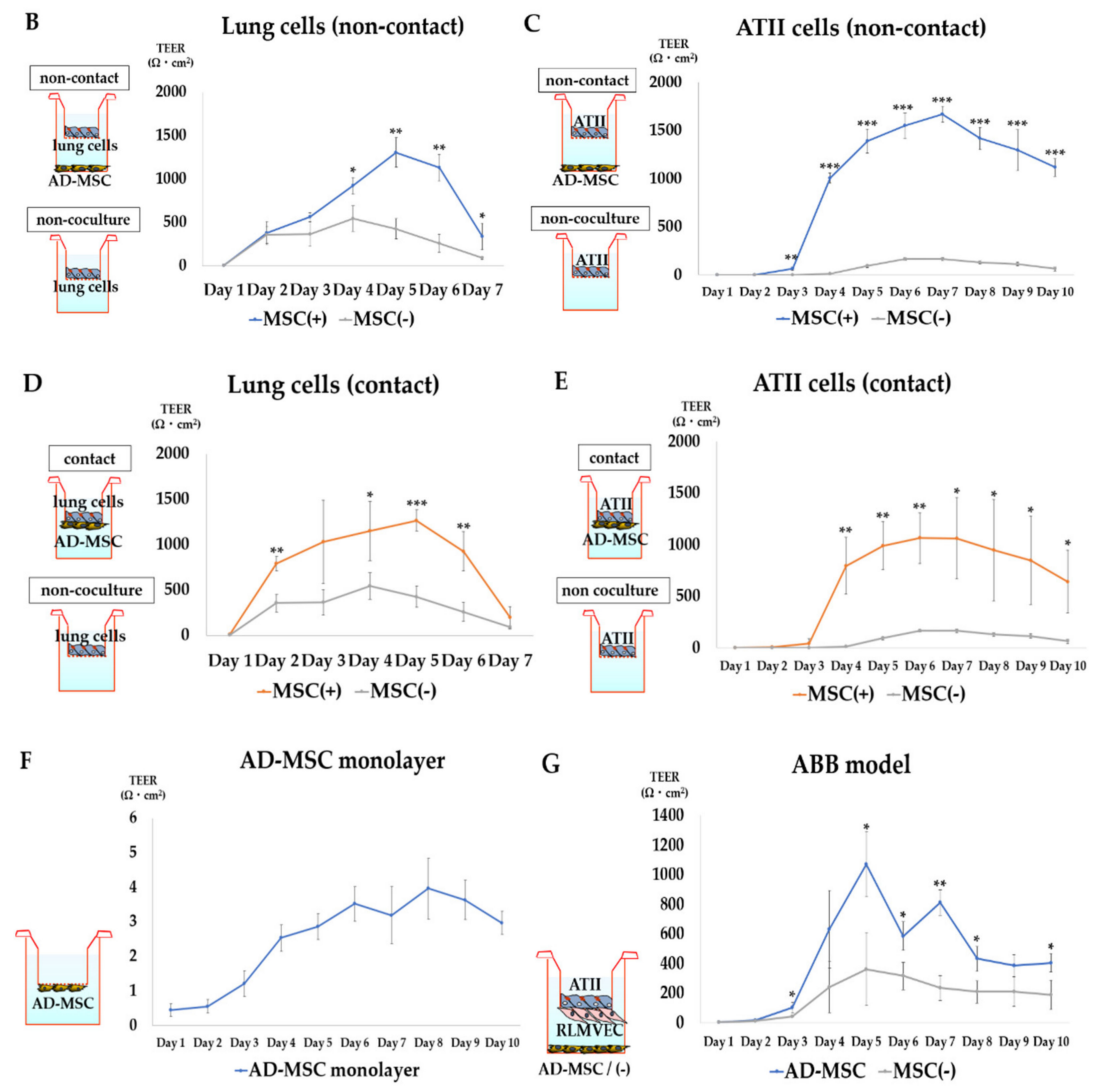
TEER measurement. (**A**) Comparison of TEER values of the lung cell monolayers by different origins of MSCs. (**B**) Comparison of TEER values between the non-contact AD-MSC coculture group and the non-coculture group in lung cell monolayers. (**C**) Comparison of TEER values between the non-contact AD-MSC coculture group and non-coculture group in ATII cell monolayers. (**D**) Comparison of TEER values between the contact AD-MSC coculture group and non-coculture group in lung cell monolayers. (**E**) Comparison of TEER values between the contact AD-MSC coculture group and non-coculture group in ATII cell monolayers. (**F**) TEER measurement of the AD-MSC monolayer. (**G**) Comparison of TEER values between the non-contact AD-MSC coculture group and non-coculture group in the air-blood barrier (ABB) model. All data represent means ± standard deviations; * *p* < 0.05, ** *p* < 0.01, *** *p* < 0.001.

First, we compared the barrier functions of AD-MSCs and BM-MSCs relative to lung cells seeded on insert membranes. The peak TEER values for the AD-MSC, BM-MSC, and non-coculture groups were 392.4 ± 131.9, 124.5 ± 21.2, and 34.8 ± 2.2 Ω·cm^2^, respectively (Figure 4A). Thus, AD-MSCs significantly enhanced the integrity of the lung cell monolayer compared with BM-MSCs.

#### 3.3.2. TEER Measurement in AD-MSC Non-Contact Culture

Based on the strong enhancement of barrier function, we selected AD-MSCs to evaluate the TEER of MSCs. We measured the peak TEER of the harvested lung cell monolayer and purified ATII cell monolayer. The peak TEER values of the lung cell monolayer for the AD-MSC non-contact group and AD-MSC non-coculture group were 1308.3 ± 170.5 and 544.5 ± 145.5 Ω cm^2^, respectively (Figure 4B). Non-contact AD-MSCs significantly enhanced the integrity of the lung cell monolayer.

The peak TEER values of the ATII cell monolayer for the AD-MSC non-contact group and AD-MSC non-coculture group were 1669.3 ± 85.5 and 167.2 ± 17.3 Ω cm^2^, respectively (Figure 4C). Non-contact AD-MSCs significantly enhanced the integrity of the ATII cell monolayer.

#### 3.3.3. TEER Measurement in AD-MSC Contact Culture

We next evaluated the effects of cell-cell contacts between AD-MSCs and lung epithelial cells on alveolar barrier function using the lung cell monolayer and ATII cell monolayer models. The peak TEER values of the lung cell monolayer for the AD-MSC contact group and AD-MSC non-coculture group were 1267.8 ± 116.5 and 544.5 ± 145.5 Ω cm^2^, respectively (Figure 4D). Contact AD-MSCs significantly enhanced the integrity of the lung cell monolayer.

The peak TEER values of the ATII cell monolayer for the AD-MSC non-contact group and AD-MSC non-coculture group were 1063.3 ± 245.9 and 167.2 ± 17.3 Ω cm^2^, respectively (Figure 4E). Non-contact AD-MSCs significantly enhanced the integrity of the ATII cell monolayer.

The TEER value of the AD-MSC monolayer was negligible (Figure 4F).

#### 3.3.4. TEER Measurement in the ABB Model

We developed an ABB model by seeding ATII cells on top of the membrane and RLMVECs on the bottom of the membrane. The peak TEER values of the bilayer of the ATII cells and RLMVECs for the AD-MSC coculture group and AD-MSC non-coculture group were 1072.1 ± 216.9 and 362.1 ± 243.1 Ω cm^2^, respectively (Figure 4G). Non-contact AD-MSCs significantly enhanced the integrity of the ABB model.

### 3.4. Permeability Assay

**Figure 5:**
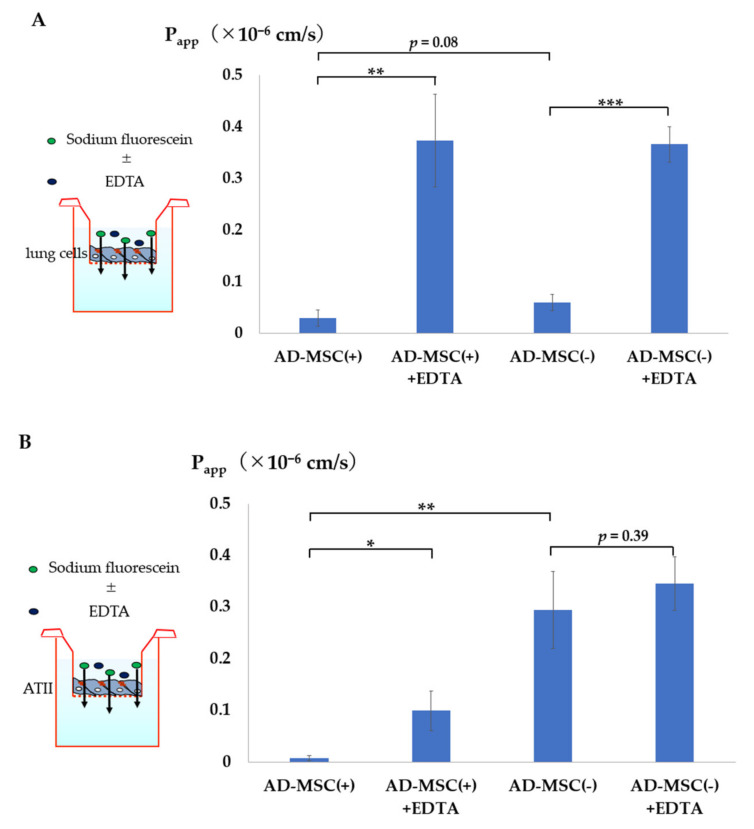
Permeability assay. (**A**) Transport of sodium fluorescein across the lung cell monolayer after 4 days of cultivation with (+) or without (−) non-contact AD-MSCs. (**B**) Transport of sodium fluorescein across the ATII cell monolayer after 7 days of cultivation with (+) or without (−) non-contact AD-MSCs. All data represent means ± standard deviations; * *p* < 0.05, ** *p* < 0.01, *** *p* < 0.001.

Using sodium fluorescein, we evaluated the microparticle permeability of the lung cell monolayer and ATII monolayer models. The Papp values of the lung cell monolayers in the AD-MSC coculture group and AD-MSC non-coculture group were 0.03 ± 0.0158 × 10^−6^ cm/s and 0.06 ± 0.0158 × 10^−6^ cm/s, respectively (Figure 5A). Moreover, the Papp values of the ATII cell monolayer for the AD-MSC coculture group and AD-MSC non-coculture group were 0.0075 ± 0.0052 × 10^−6^ cm/s and 0.294 ± 0.0748 × 10^−6^ cm/s, respectively (Figure 5B).

ATII cell monolayer analysis showed that Papp was significantly lower in the AD-MSC coculture group than in the AD-MSC non-coculture group. In the presence of EDTA, which is known to modulate tight junctions [25], the Papp value in all groups was high, reflecting the opening of the tight junctions.

### 3.5. Immunostaining and Transmission Electron Microscopy of Cell Junctions

**Figure 6:**
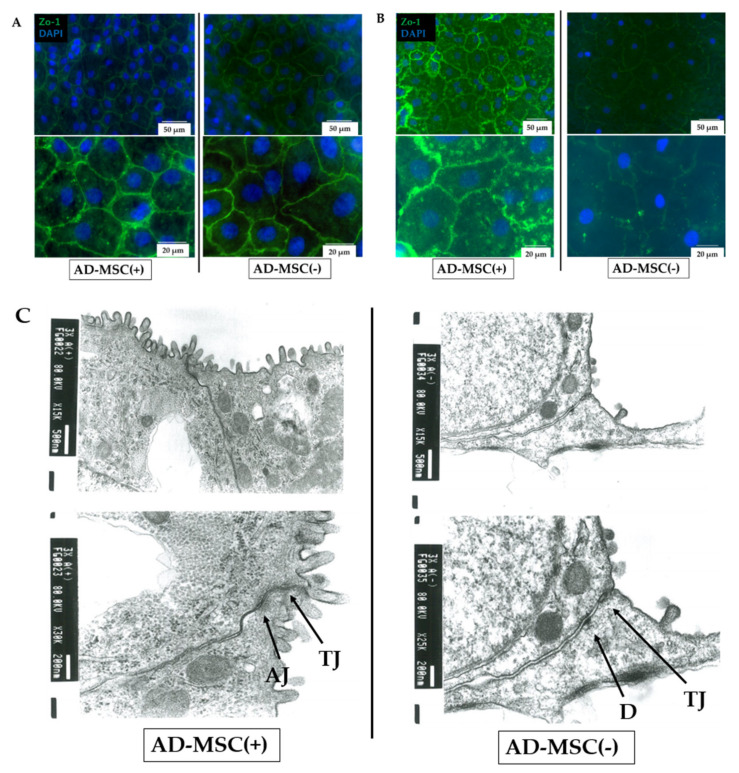
Immunostaining and transmission electron microscopy of cell junctions. (**A**) Immunostaining of lung cells seeded in insert membranes on day 4 (ZO-1). (**B**) Immunostaining of AT2 cells seeded in insert membranes on day 7 (ZO-1). (**C**) Transmission electron microscopy of ATII cells on day 7 after seeded on insert membranes and cultivation with (+) or without (−) non-contact AD-MSCs. TJ, tight junction; AJ, adherens junction; D, desmosome.

The expression of tight junctions in the membrane was assessed by analysis of ZO-1, an intracellular protein of the tight junction complex. We observed clear expression of ZO-1 at the cell boundary in the AD-MSC coculture group compared with that in the AD-MSC non-coculture group in both lung cell monolayer and ATII cell monolayer models (Figure 6A,B).

Transmission electron microscopy revealed that the AD-MSC-cocultured ATII cells formed apical junctions consisting of a tight junction and adherence junction complex. By contrast, the AD-MSC non-coculture group showed several areas in which the membranes were separated (Figure 6C).

### 3.6. PCR Analysis

Next, we evaluated 84 genes related to cell junctions; those showing more than 1.5-fold expression in the AD-MSC coculture group were listed, excluding genes whose relative expression levels were low (Table 1).

Among the genes related to tight junctions, some claudin family genes (including claudin-4), which are essential components of tight junctions, exhibited high expression levels in the AD-MSC coculture group. Additionally, many integrin family members were highly expressed in the AD-MSC coculture group.

## 4. Discussion

In 1968, MSCs were first discovered as a component of the bone marrow stromal tissue [26]. Subsequently, MSCs have been isolated from various tissues, such as adipose tissue, umbilical cord tissue, placental tissue, and exfoliated deciduous teeth [27,28,29,30,31]. Although multipotent MSCs possess robust self-renewal characteristics and the ability to differentiate into tissue-specific cells, the excellent therapeutic efficacy of MSCs for treatment of various diseases is thought to be related to MSC-derived products, such as conditioned medium (CM) and extracellular vesicles (EVs), including exosomes and microvesicles [32]. Among lung diseases, the therapeutic effects of MSCs on diseases such as asthma, chronic obstructive pulmonary disease, and ARDS have been evaluated [33,34,35], and MSCs have recently been proposed as a therapeutic option for the treatment of COVID-19 to reduce morbidity and mortality [36,37]. The main mechanism for the MSC therapeutic function is mediated by paracrine secretory factors, which have been demonstrated to induce an anti-inflammatory response, reduce apoptosis, initiate an antimicrobial innate response, protect pulmonary endothelial cells and alveolar epithelial cells from damage, and improve alveolar fluid clearance [35]. Further, MSCs can enhance the innate immune responses against bacterial infection via direct and indirect mechanisms. MSCs induce the phagocytic activity of macrophages and monocytes via promoting mitochondrial transfer [38], and MSCs also secret anti-microbial peptides and proteins, including LL-37, lipocalin-2, β-defensin-2, hepcidin, and KGF [39,40].

Although the therapeutic effects of MSCs on vascular endothelial barrier function have been analyzed, information on their effects on alveolar epithelial function is limited. In addition, the properties of MSCs vary with cell origin, because their surface phenotypes and the expression of chemokine/cytokine genes vary based on cell origins [10,11]. Therefore, in this study, we evaluated the effects of MSCs on the barrier function of alveolar epithelial cells from different perspectives in several experimental systems.

First, we compared cell origins. In TEER evaluation, a widely accepted quantitative technique to measure the integrity of tight junction dynamics in cell culture models [41,42,43], the AD-MSC coculture group exhibited a significant increase in TEER values compared with the BM-MSC coculture and non-coculture groups. In addition, AD-MSCs are relatively easy to collect, and a large number of cells can be obtained within a short period of time. Therefore, we decided to use AD-MSCs in subsequent experiments.

Second, we assessed differences in barrier function between the contact and non-contact methods. The results showed that AD-MSCs enhanced the barrier function between lung cells in both contact and non-contact cultures. Although the number of AD-MSCs seeded and the seeded area differed, making the simple comparison of data difficult, we presumed that the main effects were related to the paracrine effects of bioactive factors secreted by MSCs directly in CM or via EVs because no additive effect was observed in both AD-MSCs and lung cells/ATII cell-contact models.

Third, we confirmed the effects of AD-MSCs on barrier function using a permeability assay. Sodium fluorescein (<40 kDa) was used for the assay because exogenous macromolecules of such size are absorbed into the interstitial and vascular spaces from the air spaces through tight junctions by paracellular diffusion [44,45]. The Papp value of FluNa was very low in both the AD-MSC-added lung cell monolayer and ATII monolayer models. By contrast, without AD-MSCs, the Papp value of FluNa was significantly high in the ATII model, possibly because of the immature tight junction of ATII cells. This result was probably related to cell damage caused by cell sorting. In the presence of EDTA, which disrupts tight junctions, the Papp value of FluNa increased in both AD-MSC-added models, indicating that the effects of AD-MSCs in enhancing intercellular barrier function were counteracted.

Morphologically, ATII cells seeded on tissue culture plastic or insert membranes changed their shape to ATI-like flat cells over time. In addition, immunohistochemical analysis showed that the cells were RT1-40-positive/RT2-70-negative, suggesting that ATII cells differentiated into ATI cells, as indicated in previous reports [46,47]. Consistent with the TEER measurement and permeability assay data, intercellular fluorescence staining of the tight junction protein ZO-1 was high in AD-MSC-supplemented models. Furthermore, transmission electron microscopy showed intercellular loosening in the AD-MSC non-coculture group. Taken together, these experimental results indicate that AD-MSCs exerted major effects on the repair of the barrier function of damaged ATII cells mainly via paracrine effects.

In the current study, we analyzed the intercellular barrier function of harvested whole lung cells and sorted ATII cells. A molecular cell atlas of the human lung from single-cell RNA sequencing [48] demonstrated the anatomical locations of 58 cell populations in the human lung, suggesting that various cells may also be present in rat lungs. Although approximately 25% of the cells were epithelial cells [49], endothelial cells and institutional cells may also support the strength of intercellular tight junctions. However, our analysis of ATII cells demonstrated the presence of pure alveolar epithelial intercellular tight junctions because ATII cells differentiated into ATI cells within a short period of time, regardless of the presence of AD-MSCs. Thus, our current in vitro results demonstrated that AD-MSCs strengthened the alveolar epithelial intercellular tight junctions functionally and morphologically via paracrine effects.

Analysis of gene expression profiles of cell junctions revealed that some claudin family members, including claudin-4, which is one of the most important components of tight junctions, were highly expressed in the AD-MSC coculture group. The barrier function of epithelial cells, which generates a barrier to water and solutes, mainly involves tight junction proteins known as claudins [50]. Claudins are a family of tetraspan transmembrane proteins that form the structural basis for tight junction permeability [51,52,53]. In the lungs, the most prevalent classic claudins confirmed to be expressed throughout the respiratory epithelium are claudin-4 and -7 [54,55]. In particular, claudin-4 has been shown to promote barrier function, indicating specificity in the regulation of tight junction permeability [56,57,58,59,60].

Many integrin family members were also highly expressed in the AD-MSC coculture group in the PCR array. Integrins are cell adhesion receptors that mediate the attachment of cells to the extracellular matrix (ECM) and signal transduction from the ECM to the cells [61]. Although functional analysis was not performed in this study, MSCs may also enhance focal adhesions.

The current study had some limitations. First, recent in vitro studies have suggested that the properties of cell substrate have important effects on cell structure and function [62,63], but we did not consider the difference between the effects of insert membranes and tissue culture plastic. Second, we did not evaluate the beneficial effect of AD-MSCs in an in vitro injury model by using cytotoxic factors such as LPS. Third, we did not clarify the mechanism through which MSCs improve intercellular barrier function in the lungs. Additionally, AD-MSCs secrete various products, including cytokines, exosomes, and microvesicles. We did not analyze these MSC-derived products. Moreover, we did not demonstrate the molecular signal cascade that strengthens the intercellular barrier function. Regarding lung endothelial cells, the effects of HGF secreted from MSCs via the mammalian target of rapamycin/signal transducer and activator of transcription-3 pathway and/or Rac1 pathway on improving vascular barrier function in injured lung models was investigated [16,17]. MSCs also produce Ang-1 and keratinocyte growth factor (KGF), which enhances endothelial barrier properties [19]. With regard to the alveolar epithelium, Ang-1 restores epithelial protein permeability [64], and KGF significantly enhances barrier function by modulating the actin cytoskeleton [58]. Although cells secrete various products and have many signal cascades that affect intercellular barrier function, future big data analyses are needed to clarify these complex mechanisms.

## 5. Conclusions

In this study, TEER measurement, permeability assay, and immunohistological findings revealed that AD-MSCs enhanced the intercellular barrier function between lung epithelial cells. Although BM-MSC-based therapy has mainly been used clinically for several diseases, including ARDS, AD-MSCs may be more suitable than BM-MSCs for strengthening the barrier function of the alveolar epithelium.

## Figures and Tables

**Table 1:** Cell junction-related gene profiles.

Symbol	Description	Fold Change	Related Cell Junction
*Cav2*	Caveolin2	2.29	Focal adhesions
*Cldn15*	Claudin15	1.75	Tight junctions
*Cldn4*	Claudin4	1.89	Tight junctions
*Cldn6*	Claudin6	4.13	Tight junctions
*Dsc2*	Desmocollin2	1.81	Desmosomes
*Itga5*	Integrin, alpha 5	2.15	Focal adhesions
*Itgal*	Integrin, alpha L	1.87	Focal adhesions
*Itgam*	Integirn, alpha M	3.5	Focal adhesions
*Itgax*	Integirn, alpha X	2.78	Focal adhesions
*Itgb2*	Integrin, beta 2	1.82	Focal adhesions
*Itgb3*	Integrin, beta 3	1.89	Focal adhesions
*Itgb6*	Integrin, beta 6	2.15	Focal adhesions

## Data Availability

The data presented in this study are available from the corresponding author upon reasonable request.
